# Qingshen Buyang Formula Attenuates Renal Fibrosis in 5/6 Nephrectomized Rats via Inhibiting EMT and Wnt/*β*-Catenin Pathway

**DOI:** 10.1155/2019/5370847

**Published:** 2019-05-02

**Authors:** Xueqin Zhang, Jing Fang, Zhiqiang Chen, Bingwu Zhao, Su Wu, Yongmei Pan

**Affiliations:** Hebei University of Chinese Medicine, Shijiazhuang, Hebei 050200, China

## Abstract

As renal fibrosis significantly contributes to various kinds of chronic kidney diseases, this study aimed to investigate the renal protective effects of Qingshen Buyang Formula against renal fibrosis on 5/6 nephrectomized rats, and its underlying mechanisms were explored. A total of 24 male Sprague-Dawley rats were randomly divided into sham operation group (Sham group), 5/6 nephrectomy group (5/6Nx group), and Qingshen Buyang Formula treatment group (QBF group). The intervention was intragastric administration for 12 weeks. In the end, the blood samples were collected to test renal functional parameters, urine proteins were measured, and the left kidneys were removed for histological studies, as well as mRNA and protein expression analysis. The results showed that Qingshen Buyang Formula significantly decreased BUN, Scr, and proteinuria in 5/6Nx rats. Meanwhile, it ameliorated the kidney injury and fibrosis, exemplified by the depressed expression of collagen I and fibronectin (FN), which are the main components of ECM. Furthermore, the process of EMT inhibited the Wnt/*β*-catenin signaling pathway related genes, such as Wnt4, TCF4, *β*-catenin, and p-GSK3*β*. Collectively, the Qingshen Buyang Formula can improve renal function and attenuate renal fibrosis, and its underlying mechanisms may be related with inhibiting EMT and Wnt/*β*-catenin signaling pathway.

## 1. Introduction

Renal fibrosis is the common final pathway of diverse forms of chronic kidney diseases (CKD), which has become one of the major diseases threatening human health [1, 2]. Renal fibrosis is characterized as excessive deposition of extra cellular matrix (ECM), including fibronectin and collagens [3]. Previous studies have suggested that renal fibrosis correlates with the progression to end-stage renal diseases [4]. Thus, strategies that can prevent renal fibrosis could have a marked clinical influence [5]. However, until now, there are still few clinical treatment options [6].

Qingshen Buyang Formula is prescribed based on the theory of traditional Chinese medicine, which is the most important and widely used therapy for various diseases in China for thousands of years. The formula contains 14 natural herbs and has the function of promoting blood stasis, eliminating turbid dampness, and tonifying YangQi. It has been used in clinic for years to treat various kinds of chronic kidney diseases and gained satisfactory renal protective effects. Yet, the underlying mechanisms have not been fully explored.

Renal fibrosis has a very complicated etiology and pathogenesis. Among them, EMT and Wnt/*β*-catenin pathway are frequently studied. EMT of tubular epithelial cells is an important mechanism involved in renal fibrosis [7]. The contribution of EMT to renal fibrosis has been described to occur in various fibrotic kidney diseases [8]. It is believed that, through EMT, the injured tubular epithelial cells induce accumulation of fibroblasts or myofibroblasts and the abundant deposition of ECM, ultimately leading to renal fibrosis [9]. Wnt/*β*-catenin signaling pathway is an evolutionarily conserved, developmental pathway implicated in regulating a variety of biological processes, such as cell fate determination, tissue homeostasis, and organ development [10]. Although Wnt/*β*-catenin pathway is quiescent in normal adult kidney, it can be reactivated in various models of kidney injury. Sustained activation of Wnt/*β*-catenin pathway is detrimental and could lead to renal fibrosis. Several comprehensive reviews have highlighted the crucial role of this pathway [11, 12].

In this study, we use the 5/6 nephrectomized rats model to simulate the process of chronic kidney disease, so as to observe the effects of Qingshen Buyang Formula on kidney injury and fibrosis and explore its mechanisms based on EMT and Wnt/*β*-catenin pathway.

## 2. Materials and Methods

### 2.1. Animals

A total of 24 male Sprague-Dawley rats (8 weeks old, weighting 180~200 g) were purchased from Beijing Vital River Laboratory Animal Technology Co., Ltd. (SPF grade, license number: SCXK (Jing) 2012-0001). All animals were reared in a light-controlled (12 h/12 h light/dark cycle) room at 24±1°C and humidity of 50%-70% and given free access to standard rat chow and water. The study was approved by the Ethics Committee of Hebei University of Chinese Medicine. Every effort was made to minimize suffering.

### 2.2. Animal Modeling and Grouping

After being acclimated for 1 week, the rats were randomly divided into 3 groups (n = 8 per group), namely, sham operation group (Sham group), 5/6 nephrectomy group (5/6Nx group), and Qingshen Buyang Formula treatment group (QBF group). Using full sterile technique, all rats underwent sham operation or 5/6 nephrectomy under anesthesia with 10% chloral hydrate (3 ml/kg). 5/6 nephrectomy was performed using a standard two-step surgical ablation procedure [13, 14]. First, 2/3 of the left kidney was removed by amputation of both renal poles. Then, 1 week later, the right kidney was totally removed, while the Sham group, serving as control group, underwent a sham operation without damage to the kidney.

### 2.3. Drug Administration

Two weeks after the second surgery, the rats of QBF group were administrated with Qingshen Buyang herbal granules at a dosage of 3.24 g/kg daily, and those of Sham group and 5/6Nx group were fed with equal volume of drinking water (Figure S1).

Herbs of Qingshen Buyang Formula were provided by Guangdong Yifang Pharmaceutical Co., Ltd. (Foshan, China). The herbs are composed of 14 Chinese Materia Medica (CMMs): Huangqi (*Radix Astragali*), Danggui (*Radix Angelicae Sinensis*), Chuanxiong (*Rhizoma Chuanxiong*), Honghua (*Flos Carthami*), Dilong (*Pheretima*), Shuizhi (*Hirudo*), Xianmao (*Rhizoma*), Xianlingpi (*Herba Epimedii*), Huoxiang (*Herba Pogostemonis*), Peilan (*Herba Eupatorii*), Chenpi (*Pericarpium Citri Reticulatae*), Doukou (*Fructus Cardamoni*), Tufuling (*Rhizoma Smilacis Glabrae*), and Jixuecao (*Centella Asiatica L.*), with a weight ratio of 30: 15: 12: 10: 12: 6: 12: 15: 10: 10: 15: 10: 30: 30.

### 2.4. Sample Collection

After 12 weeks of intervention, 24h urine was collected in metabolic cages, and then the rats were sacrificed and the blood samples were collected from abdominal aorta. The left kidneys were removed and spliced into several parts. Some of them were fixed in 4% paraformaldehyde for histological studies, and others were stored in liquid nitrogen for protein and mRNA extractions.

### 2.5. Renal Functional Parameters Test

The blood urea nitrogen (BUN) and serum creatinine (Scr) were tested by a Hitachi automatic biochemical analyzer (7600-020, Hitachi, Tokyo, Japan).

### 2.6. 24h Urine Analysis

The urine volume of each rat was recorded, and the urine proteins were tested by a Hitachi automatic biochemical analyzer (7600-020, Hitachi, Tokyo, Japan).

### 2.7. Renal Histopathology

The paraffin-embedded kidney sections (thickness 2 *μ*m) were stained with hematoxylin-eosin (HE), periodic acid-Schiff (PAS), Masson trichrome, and Picrosirius red. The operation of staining the sections was according to the instructions. Morphology of kidney tissue was observed under BX63 + DP72 optical microscope (Olympus Co., Ltd., Tokyo, Japan). Renal lesions areas in HE staining pictures and renal fibrosis areas in Masson staining pictures were quantified using Image-Pro Plus 6.0 image analysis system.

### 2.8. Immunohistochemistry Studies

After deparaffinization, the sections were incubated in 3% H_2_O_2_ deionized water and then the epitopes were retrieved by microwave. Next, the sections were blocked with goat serum and incubated with primary antibodies overnight at 4°C. Afterwards, incubation of biotinylated goat secondary antibody and streptavidin-HRP was conducted, followed by diaminobenzidine (DAB) staining, hematoxylin counterstaining, and gummy neutral balsam. Finally, the sections were imaged using the microscope. The primary antibodies used are as follows: E-Cadherin (GTX100443, GeneTex Inc., CA, USA, 1: 500), *ɑ*-SMA (ET1607-43, Hangzhou HuaAn Biotechnology Co., Ltd., Hangzhou, China, 1: 200), Vimentin (ab92547, Abcam, Inc., UK, 1: 400), and FN (ab2413, Abcam, Inc., UK, 1: 300).

### 2.9. Immunofluorescent Analysis

For immunofluorescence staining, the sections were blocked with goat serum and incubated with E-Cadherin (1: 200) and *ɑ*-SMA (1: 100) antibodies and collagen I (AF7001, Affinity Biosciences, Cincinnati, OH, USA, 1: 200) at 4°C overnight. Then the specimens were incubated with DAPI and secondary antibodies. Finally, the reaction was visualized using a fluorescence microscope.

### 2.10. Western Blot Analysis

Total protein was extracted from the renal cortex of rats and quantified with the BCA assay (MultiSciences Biotech Co., Ltd., Hangzhou, China). Then the protein was diluted with 5× loading buffer and denatured at 100°C for 5 minutes. Afterwards, the protein samples were electrophorised in SDS polyacrylamide gel and then transferred onto PVDF membranes (Millipore Corporation, Bedford, MA, USA). The nonspecific background bindings were blocked with 5% nonfat milk for an hour. Then the membranes were incubated in primary antibodies at 4°C overnight and secondary antibodies for 1 hour. Optical density of the bands was scanned by Odyssey infrared fluorescence imaging system (LI-COR, USA) and quantified using ImageJ (National Institutes of Health, USA). The primary antibodies used are as follows: Wnt4 (sc-376279, Santa Cruz Biotechnology, Inc., USA, 1: 500), TCF4 (sc-166699, Santa Cruz Biotechnology, Inc., USA, 1: 500), GSK3*β* (ab93926, Abcam, Inc., UK, 1: 1000), p-GSK3*β* (ab131097, Abcam, Inc., UK, 1: 1000), E-Cadherin (GTX100443, GeneTex Inc., CA, USA, 1: 1000), *ɑ*-SMA (ET1607-43, Hangzhou HuaAn Biotechnology Co., Ltd., Hangzhou, China, 1: 5000), and Vimentin (ab92547, Abcam, Inc., UK, 1: 5000).

### 2.11. Quantitative Real-Time Polymerase Chain Reaction (qPCR) Assay

Total RNA was extracted from kidney tissue with the TRIzol Reagent (Life Technologies Corporation, Carlsbad, CA, USA). Its concentrations and purity were tested by Nanodrop 2000C spectrophotometer (Thermo Scientific, Wilmington, DE, USA). Then the RNA was reverse-transcribed into cDNA by RevertAid First Strand cDNA Synthesis Kit (Thermo, #K1622). Gene-specific primers (see Table 1) were designed and synthesized by Wuhan Servicebio Technology Co., Ltd. (Wuhan, China). Polymerase chain reactions were performed with Platinum5 SYBR5 Green qPCR Super Mix-UDG (Invitrogen Co., Carlsbad, CA, USA) on Eco Real-Time PCR System (Illumina, San Diego, CA, USA). Each reaction was carried out in triplicate. And the relative expression amounts were calculated by the 2^−ΔΔCT^ method.

### 2.12. Statistical Analysis

The statistical analysis was performed using SPSS software (version 19.0, IBM, Chicago, IL, USA). The results were presented as mean ± standard deviation (SD). One-way ANOVA was used for intergroup comparisons and SNK-q test for the evaluation of differences between two groups.* P*< 0.05 was considered statistically significant.

## 3. Results

### 3.1. Effects of Qingshen Buyang Formula on Renal Functional Parameters of Rats

At the end of 12 weeks after surgery, the renal functional parameters, BUN and Scr, were tested to determine the effects of Qingshen Buyang Formula on renal function. As shown in Table 2, the 5/6 nephrectomized rats had obviously increased BUN and Scr levels compared with the sham operation rats (*P *< 0.05). Additionally, the BUN, Scr, and 24h UTP in rats treated with Qingshen Buyang Formula were significantly decreased compared to the 5/6 nephrectomized rats (*P*< 0.05). Thus, we conclude that Qingshen Buyang Formula can protect renal function by decreasing BUN and Scr.

### 3.2. Effects of Qingshen Buyang Formula on Proteinuria

As shown in Table 3, there is no significant difference in urine volume among the three groups. Meanwhile, 24h urinary total protein (24h UTP) and 24h urinary microalbumin (24h UMA) in the 5/6Nx group were significantly higher than those in the Sham group. Notably, 24h UTP and UMA in the QBF group were obviously lower compared with 5/6Nx group. Collectively, Qingshen Buyang Formula can decrease proteinuria.

### 3.3. Effects of Qingshen Buyang Formula on Renal Histopathology

**Figure 1:**
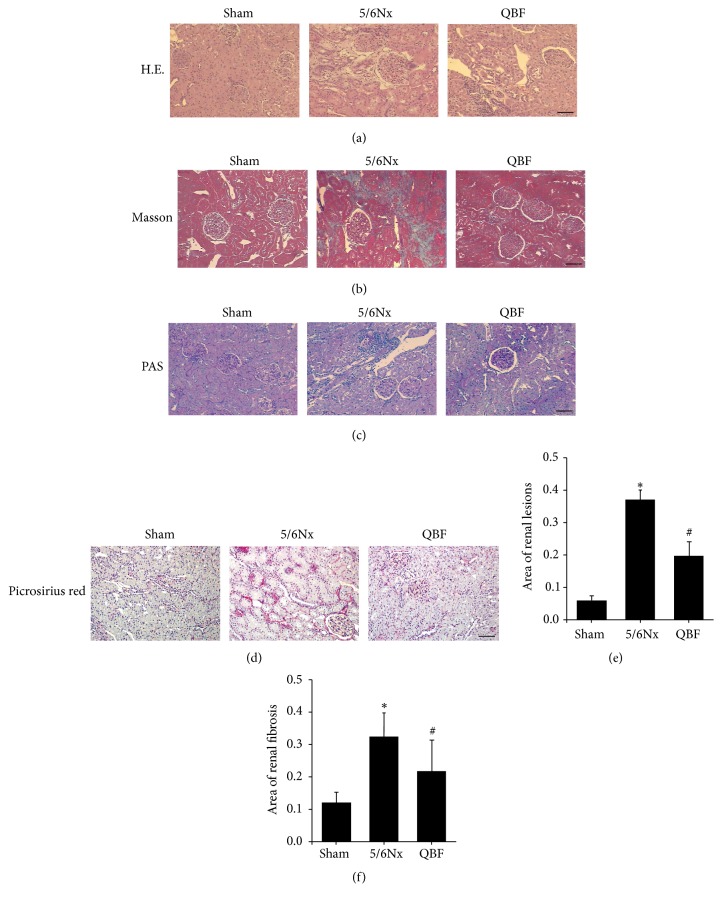
The treatment with Qingshen Buyang Formula attenuated renal lesions and fibrosis caused by 5/6 nephrectomy. ((a)–(d)) Kidney sections of the three groups were subjected to HE staining, Masson staining, PAS staining, and Picrosirius red staining. Representative micrographs showed that Qingshen Buyang Formula ameliorated kidney injury characterized by segmental sclerosis of glomeruli, focal renal fibrosis, tubular epithelial cells vacuolization, tubular dilatation, tubular atrophy, and infiltration of inflammatory cells. Scale bar, 50 *μ*m.

Under light microscopy with HE staining (Figure 1(a)), Masson trichrome staining (Figure 1(b)), PAS staining (Figure 1(c)), and Picrosirius red staining (Figure 1(d)), rats of sham operation group showed normal morphology, and no significant changes were found. In contrast, 5/6 nephrectomized rats showed segmental sclerosis of remnant glomeruli, focal renal fibrosis, enlargement of tubular lumen, tubular epithelial cells vacuolization, tubular atrophy, interstitial expansion, and numerous infiltration of inflammatory cells. Notably, Qingshen Buyang Formula treatment significantly attenuated renal lesions (Figure 1(e)) and renal fibrosis (Figure 1(f)) caused by the 5/6 nephrectomy, as compared to the untreated 5/6 nephrectomized rats. Taken together, Qingshen Buyang Formula can ameliorate renal histopathological changes.

### 3.4. Effects of Qingshen Buyang Formula on Renal Fibrosis Related Proteins

**Figure 2:**
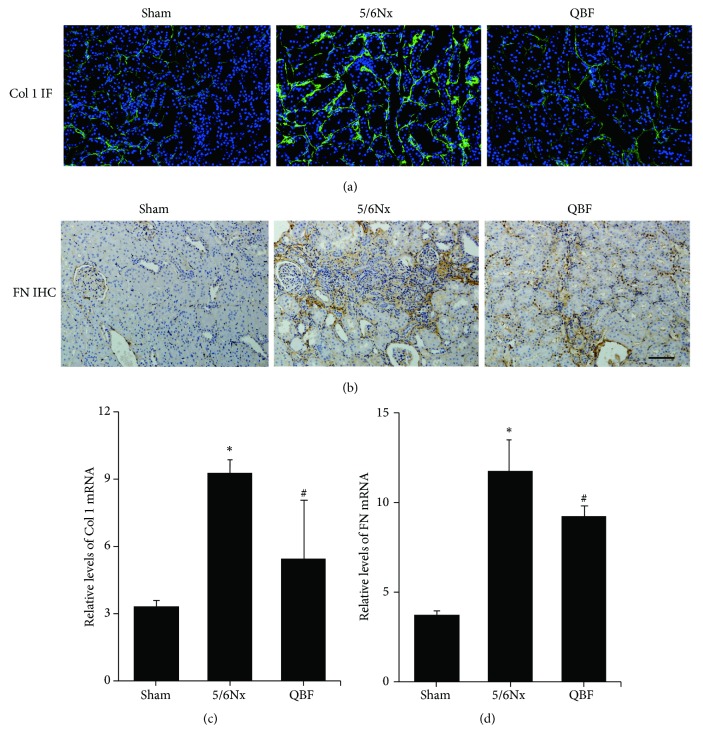
Qingshen Buyang Formula decreased the expression of collagen I and FN. ((a) and (b)) Immunofluorescence and immunohistochemistry staining demonstrated that the protein expression of collagen I and FN, which are the most important components of ECM, was inhibited by Qingshen Buyang Formula. Scale bar, 50*μ*m. ((c) and (d)) Graphic presentation of mRNA expression of collagen I and FN demonstrated significant difference among the three groups. *∗ P*< 0.05, compared with the Sham group. #* P*< 0.05, compared with the 5/6Nx group. The error bars represent the standard deviation of measurements for 8 particles in three separate sample runs (n = 24).

Collagen I and FN, which are well known as important components of ECM, are closely related to renal fibrosis. Immunofluorescence and immunohistochemistry staining showed that collagen I (Figure 2(a)) and FN (Figure 2(b)) staining were significantly increased in the 5/6Nx group compared to the sham operation group, while treatment with Qingshen Buyang Formula markedly alleviated these effects. Consistent with this result, Qingshen Buyang Formula evidently inhibited the mRNA expression of collagen I (Figure 2(c)) and FN (Figure 2(d)) in contrast to the 5/6Nx group (*P*< 0.05). Therefore, it can be concluded that Qingshen Buyang Formula attenuates renal fibrosis by inhibiting ECM deposition.

### 3.5. Effects of Qingshen Buyang Formula on EMT

**Figure 3:**
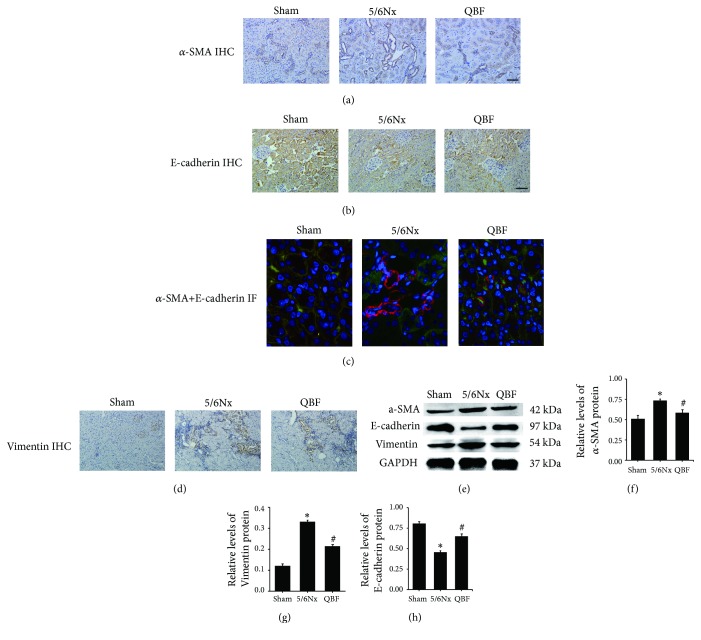
Qingshen Buyang Formula reduced EMT induced by 5/6 nephrectomy. (a) Representative micrographs showed that the upexpression of *ɑ*-SMA in 5/6Nx group was downregulated by Qingshen Buyang Formula. Scale bar, 50 *μ*m. (b) Representative micrographs showed that the downexpression of E-cadherin in 5/6Nx group was increased by Qingshen Buyang Formula. Scale bar, 50 *μ*m. (c) Immunofluorescence showed that Qingshen Buyang Formula inhibited the overexpresssion of *ɑ*-SMA and simultaneously promoted the expression of E-cadherin. Scale bar, 20 *μ*m. (d) Representative micrographs showed that the upexpression of Vimentin in 5/6Nx group was downregulated by Qingshen Buyang Formula. Scale bar, 50 *μ*m. (e) Representative western blot analyses demonstrated that *ɑ*-SMA and Vimentin were overexpressed and E-cadherin was depressed in 5/6Nx group, which was reversed by Qingshen Buyang Formula. ((f)–(h)) Graphic presentation of western blot analyses in the three groups as indicated. *∗ P*< 0.05, compared with the Sham group. #* P*< 0.05, compared with the 5/6Nx group. The error bars represent the standard deviation of measurements for 8 particles in three separate sample runs (n = 24).

EMT is characterized by downregulation of E-cadherin and upregulation of some mesenchymal genes, typically *ɑ*-SMA and Vimentin. To examine the effect of Qingshen Buyang Formula on EMT, we tested the expression of E-cadherin, *ɑ*-SMA, and Vimentin by immunohistochemistry, immunofluorescence, and western blot. Immunohistochemistry staining of *ɑ*-SMA (Figure 3(a)), E-cadherin (Figure 3(b)), and Vimentin (Figure 3(d)) showed that 5/6 nephrectomy induced upregulation of *ɑ*-SMA and Vimentin expression and downregulation of E-cadherin expression. Notably, the expression of *ɑ*-SMA and Vimentin was remarkably depressed, while the expression of E-cadherin was prominently promoted when the 5/6 nephrectomized rats were treated with Qingshen Buyang Formula. The results of immunofluorescence (Figure 3(c)) and western blot (Figures 3(e), 3(f), 3(g), and 3(h)) are in accordance with that. Thus, we conclude that Qingshen Buyang Formula restrains EMT.

### 3.6. Effects of Qingshen Buyang Formula on the Wnt/*β*-Catenin Signaling Pathway

**Figure 4:**
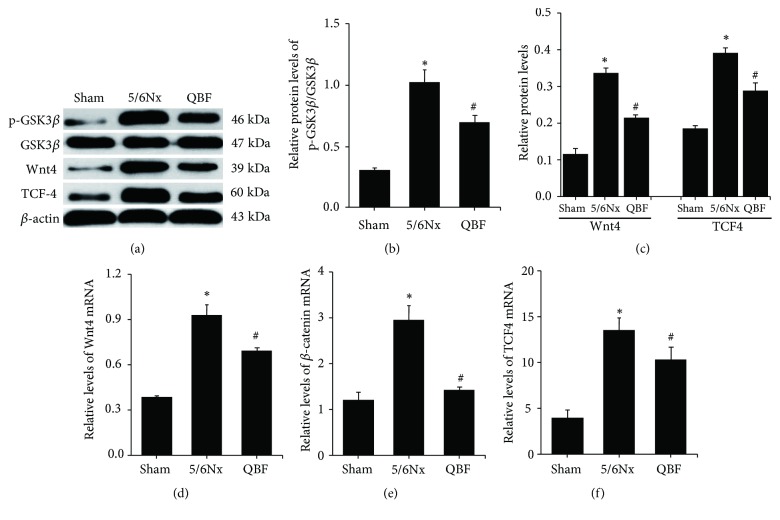
Qingshen Buyang Formula inhibited activated Wnt/*β*-catenin signaling pathway. (a) Representative western blot analyses illustrated that Qingshen Buyang Formula suppressed the protein expression of Wnt4, TCF4, and p-GSK3*β* involved in Wnt/*β*-catenin signaling pathway, while the expression of GSK3*β* had no change. ((b) and (c)) Graphic presentation of Wnt4, TCF4, and p-GSK3*β*/GSK3*β* in the three groups as indicated. ((d)–(f)) Real-time PCR revealed that Qingshen Buyang Formula reversed the increased mRNA expression of Wnt4, TCF4, and *β*-catenin. *∗ P*< 0.05, compared with the Sham group. #* P*< 0.05, compared with the 5/6Nx group. The error bars represent the standard deviation of measurements for 8 particles in three separate sample runs (n = 24).

Wnt signaling has been reported to be hyperactive and detrimental in the progress of renal fibrosis. Based on this, we further examined the expression of Wnt signaling molecules by western blot and qPCR. The western blot results (Figures 4(a), 4(b), and 4(c)) demonstrated that the protein expressions of Wnt4, TCF4, and p-GSK3*β* in 5/6Nx group were obviously higher than those in sham operation group (*P*< 0.05), indicating the activation of Wnt/*β*-catenin signaling pathway. Of note, Qingshen Buyang Formula significantly decreased the protein expression of Wnt4, TCF4, and p-GSK3*β*, whereas the expression of GSK3*β* in each group had no difference. In accordance with the western analysis, the qPCR (Figures 4(d), 4(e), and 4(f)) also confirmed that the Wnt/*β*-catenin signaling was overactivated in 5/6Nx rats. Meanwhile, Qingshen Buyang Formula could inhibit the overexpression of Wnt4, TCF4, and *β*-catenin mRNA expression (*P *< 0.05). Collectively, we conclude that Qingshen Buyang Formula may inhibit the overactiviation of Wnt/*β*-catenin signaling pathway.

## 4. Discussion

Renal fibrosis is the major hallmark of various types of progressive chronic kidney diseases. Aberrant and excessive deposition of ECM is the typical characteristic of renal fibrosis and amplifies the severity of kidney injury. In the present study, renal protective effects of Qingshen Buyang Formula on 5/6 nephrectomized rats were evaluated. The results demonstrate that Qingshen Buyang Formula improves renal function, decreases proteinuria, and ameliorates 5/6 nephrectomy-induced kidney injury, fibrosis, and deposition of ECM. Further study also reveals that the underlying mechanisms in the process are related to the inhibitory effect of the herbs on EMT and Wnt/*β*-catenin signaling pathway.

According to the theory of traditional Chinese medicine, the pathogenesis of renal fibrosis mainly focused on turbid dampness and blood stasis, accompanied with YangQi deficiency. In this point of view, Qingshen Buyang Formula is prescribed as basic medicine to treat renal fibrosis. The Qingshen Buyang herbs used in this study can be categorized into 3 types based on their therapeutic functions.* Radix Angelicae Sinensis*,* Rhizoma Chuanxiong*,* Flos Carthami*,* Pheretima*, and* Hirudo *can promote blood circulation and dredge collaterals, thus removing blood stasis.* Herba Pogostemonis*,* Herba Eupatorii*,* Pericarpium Citri Reticulatae*,* Fructus Cardamoni*,* Rhizoma Smilacis Glabrae*, and* Centella Asiatica *L. together have the function of eliminating turbid dampness.* Radix Astragali*,* Rhizoma*, and* Herba Epimedii* cooperate to tonify Yang Qi. Collectively, the 14 Qingshen Buyang herbs can clear away pathological factors including blood stasis and turbid dampness on the basis of nourishing YangQi.

Qingshen Buyang Formula has exhibited significant renal protective effects in clinical treatment and earlier studies suggest that these herbs can alleviate renal fibrosis by inhibiting the expression of fibronectin, laminin, and TGF-*β*1 [15, 16]. In accordance with this, the results of this study showed that Qingshen Buyang Formula can protect renal function by decreasing BUN, Scr, and proteinuria compared with the 5/6Nx group. At the same time, the herbs can relieve the pathological changes caused by 5/6 nephrectomy. Besides, the renal fibrosis and ECM deposition were significantly alleviated with a reduction of collagen I and fibronectin.

The concept of EMT is first described by Elizabeth Hay in the late 1960s. Since then, many novel findings have been published. The process of complete EMT can be artificially divided into four steps. The first and pivotal step is the downregulation of E-cadherin [17]. Once E-cadherin is downregulated, the epithelium loses cell-to-cell junctions [18]. Next, *α*-SMA expression is induced and actin reorganized [19]. Then the transitioning cell changes into a mesenchymal phenotype [20]. Recently, Lovisa et al. [21] and Grande et al. [22] proposed new concept of partial EMT. They found that, after injury, TECs got the phenotype change* in situ *without leaving renal tubules. Although EMT is a controversial subject, a consensus that EMT plays a significant role in renal fibrosis is reached [9, 23]. In this study, 5/6 nephrectomy successfully induced tubular EMT, while the treatment with Qingshen Buyang Formula prominently alleviated the process by downregulation of *ɑ*-SMA and upregulation of E-Cadherin. Therefore, the renal protective effects of Qingshen Buyang Formula may be related to inhibiting EMT.

To date, Wnt/*β*-catenin signaling pathway has attracted much attention for its function to promote renal fibrosis. The Wnt ligands, by binding to their receptors, can transmit their signal across the plasma membrane and inhibit the *β*-catenin destruction complex [24]. Then the stabilized *β*-catenin translocates into the nuclei and binds to TCF/LEF to stimulate the transcription of Wnt target genes [25, 26]. Accumulating studies have demonstrated that activation of Wnt/*β*-catenin signaling promotes renal fibrosis in various models of kidney injury [27–29]. In line with this finding, inhibition of Wnt/*β*-catenin signaling is associated with significantly improved outcomes [30–33]. In the present study, Wnt/*β*-catenin signaling is overactivated by 5/6 nephrectomy, reflecting as the markedly increasing protein and mRNA expression levels of Wnt4, TCF4, p-GSK3*β*, and *β*-catenin. What is more, the Qingshen Buyang Formula lowers these indicators. In all, the results suggest that the renal protective effects of Qingshen Buyang Formula may be related to inhibiting Wnt/*β*-catenin signaling pathway.

There are still some limitations to our study. First, Qingshen Buyang Formula consists of multiple drugs, while the actual effective components and their interactions are unclear until now. So further studies are needed to figure out the effective components in the formula, and every effective component should be tested as drug control. Second, experiments in vitro are needed to reconfirm the directly inhibiting effects of the formula on activation of Wnt/*β*-catenin and Wnt/*β*-catenin-induced EMT. Therefore, we will explore these problems further and in depth to make the experiment more precise and meaningful.

## 5. Conclusion

In summary, our study demonstrates that Qingshen Buyang Formula can improve renal function, ameliorate renal fibrosis, and reduce ECM deposition. Furthermore, the underlying mechanisms may be related to inhibiting EMT and Wnt/*β*-catenin signaling pathway. Thus, the Qingshen Buyang Formula may be an alternative therapy for renal fibrosis.

## Figures and Tables

**Table 1:** Specific primers used in qPCR.

Genes	Forward primers (5′→3′)	Reverse primers (3′→5′)	Length of product (bp)
GAPDH	CTGGAGAAACCTGCCAAGTATG	GGTGGAAGAATGGGAGTTGCT	138
Wnt4	GGAAGGTGGTGACACAAGGGA	CATAGGCGATGTTGTCCGAGC	189
TCF4	TGGAGACGCTCTGGGGAAAG	CAACAGGAGTTGAAGGGTTGGA	89
*β*-catenin	ATTACGACAGACTGCCTTCAGATC	GAGCAGACAGACAGCACCTTCA	167

**Table 2:** Renal function parameters of different groups.

Group	Blood urea nitrogen (mmol/L)	Serum creatinine (*μ*mol/L)
Sham	5.71±0.46	44.09±7.59
5/6Nx	17.79±1.98^*∗*^	66.77±3.35^*∗*^
QBF	11.61±3.02^#^	58.19±8.99^#^

Notes. Compared with Sham group, ^*∗*^P < 0.05; ^#^compared with 5/6Nx group, P < 0.05.

**Table 3:** Proteinuria in each group.

Group	Urine volume (ml)	24h UTP (mg/d)	24h UMA (mg/d)
Sham	13.81±4.54	9.02±2.91	0.10±0.02
5/6Nx	17.37±4.24	13.70±2.97^*∗*^	0.33±0.12^*∗*^
QBF	11.12±3.20	8.22±2.46^#^	0.17±0.04^#^

Notes. Compared with Sham group, ^*∗*^*P*< 0.05; compared with 5/6Nx group, ^#^*P*< 0.05.

## Data Availability

The data used to support the findings of this study are available from the corresponding author upon request.

## Supplementary Materials

Supplementary Materials Figure S1: experimental design diagram.

## References

[1] Couser W. G., Remuzzi G., Mendis S., Tonelli M. (2011). The contribution of chronic kidney disease to the global burden of major noncommunicable diseases. *Kidney International*.

[2] Nugent R. A., Fathima S. F., Feigl A. B., Chyung D. (2011). The burden of chronic kidney disease on developing nations: a 21st century challenge in global health. *Nephron Clinical Practice*.

[3] Boor P., Ostendorf T., Floege J. (2010). Renal fibrosis: novel insights into mechanisms and therapeutic targets. *Nature Reviews Nephrology*.

[4] Farris A. B., Colvin R. B. (2012). Renal interstitial fibrosis: mechanisms and evaluation. *Current Opinion in Nephrology and Hypertension*.

[5] Tonelli M., Wiebe N., Culleton B. (2006). Chronic kidney disease and mortality risk: a systematic review. *Journal of the American Society of Nephrology*.

[6] Grams M. E., Chow E. K. H., Segev D. L., Coresh J. (2013). Lifetime Incidence of CKD stages 3-5 in the United States. *American Journal of Kidney Diseases*.

[7] Iwano M., Plieth D., Danoff T. M., Xue C., Okada H., Neilson E. G. (2002). Evidence that fibroblasts derive from epithelium during tissue fibrosis. *The Journal of Clinical Investigation*.

[8] Inoue T., Umezawa A., Takenaka T., Suzuki H., Okada H. (2015). The contribution of epithelial-mesenchymal transition to renal fibrosis differs among kidney disease models. *Kidney International*.

[9] Liu Y. (2010). New insights into epithelial-mesenchymal transition in kidney fibrosis. *Journal of the American Society of Nephrology*.

[10] Yongping W., Zhou Chengji J., Youhua L. (2018). Wnt signaling in kidney development and disease. *Progress in Molecular Biology and Translational Science*.

[11] Zhou D., Tan R. J., Fu H., Liu Y. (2016). Wnt/*β*-catenin signaling in kidney injury and repair: a double-edged sword. *Laboratory Investigation*.

[12] Tan R. J., Zhou D., Zhou L., Liu Y. (2014). Wnt/*β*-catenin signaling and kidney fibrosis. *Kidney International Supplements*.

[13] van Koppen A., Verhaar M. C., Bongartz L. G., Joles J. A. (2013). 5/6th nephrectomy in combination with high salt diet and nitric oxide synthase inhibition to induce chronic kidney disease in the Lewis rat.. *Journal of visualized experiments : JoVE*.

[14] Wu-Wong J. R., Chen Y.-W., Wessale J. L. (2015). Vitamin D receptor agonist VS-105 improves cardiac function in the presence of enalapril in 5/6 nephrectomized rats. *American Journal of Physiology-Renal Physiology*.

[15] Yongmei P., Zhiqiang C. (2016). Effects of huoxue huayu decoction on kidney interstitial fibrosis in diabetic nephropathy rats model. *JETCM*.

[16] Chunyu C., Zhiqiang C., Jing F. (2015). Effect of Huayu Tongluo traditional Chinese medicine on kidney fibronectin and laminin expression of diabetic nephropathy rats. *Natural Product Research and Development*.

[17] Lamouille S., Xu J., Derynck R. (2014). Molecular mechanisms of epithelial-mesenchymal transition. *Nature Reviews Molecular Cell Biology*.

[18] Kovacic J. C., Mercader N., Torres M., Boehm M., Fuster V. (2012). Epithelial-to-mesenchymal and endothelial-to-mesenchymal transition from cardiovascular development to disease. *Circulation*.

[19] Saito A. (2013). EMT and EndMT: regulated in similar ways?. *The Journal of Biochemistry*.

[20] Rastaldi M. P., Ferrario F., Giardino L. (2002). Epithelial-mesenchymal transition of tubular epithelial cells in human renal biopsies. *Kidney International*.

[21] Lovisa S., LeBleu V. S., Tampe B. (2015). Epithelial-to-mesenchymal transition induces cell cycle arrest and parenchymal damage in renal fibrosis. *Nature Medicine*.

[22] Grande M. T., Sánchez-Laorden B., López-Blau C. (2015). Snail1-induced partial epithelial-to-mesenchymal transition drives renal fibrosis in mice and can be targeted to reverse established disease. *Nature Medicine*.

[23] Wing M. R., Ramezani A., Gill H. S., Devaney J. M., Raj D. S. (2013). Epigenetics of progression of chronic kidney disease: fact or fantasy?. *Seminars in Nephrology*.

[24] Clevers H., Nusse R. (2012). Wnt/*β*-catenin signaling and disease. *Cell*.

[25] White B. D., Nguyen N., Moon R. (2007). Wnt signaling: it gets more humorous with age. *Current Biology*.

[26] MacDonald B. T., Semenov M. V., He X. (2007). SnapShot: Wnt/*β*-catenin signaling. *Cell*.

[27] He W., Dai C., Li Y., Zeng G., Monga S. P., Liu Y. (2009). Wnt/beta-catenin signaling promotes renal interstitial fibrosis. *Journal of the American Society of Nephrology*.

[28] Von Toerne C., Schmidt C., Adams J. (2009). Wnt pathway regulation in chronic renal allograft damage. *American Journal of Transplantation*.

[29] Nelson P. J., Von Toerne C., Gröne H.-J. (2011). Wnt-signaling pathways in progressive renal fibrosis. *Expert Opinion on Therapeutic Targets*.

[30] Matsuyama M., Nomori A., Nakakuni K., Shimono A., Fukushima M. (2014). Secreted frizzled-related protein 1 (Sfrp1) regulates the progression of renal fibrosis in a mouse model of obstructive nephropathy. *The Journal of Biological Chemistry*.

[31] Xue H., Xiao Z., Zhang J. (2013). Disruption of the Dapper3 gene aggravates ureteral obstruction-mediated renal fibrosis by amplifying Wnt/*β*-catenin signaling. *The Journal of Biological Chemistry*.

[32] Chen B., Dodge M. E., Tang W. (2009). Small molecule-mediated disruption of Wnt-dependent signaling in tissue regeneration and cancer. *Nature Chemical Biology*.

[33] Zhou L., Li Y., Hao S. (2015). Multiple genes of the renin-angiotensin system are novel targets of Wnt/*β*-catenin signaling. *Journal of the American Society of Nephrology*.

