# Supplementary figures and images for: Qingshen Buyang Formula Attenuates Renal Fibrosis in 5/6 Nephrectomized Rats via Inhibiting EMT and Wnt/β-Catenin Pathway
Source: Evid Based Complement Alternat Med. 2019 May 2;2019:5370847. doi: 10.1155/2019/5370847 (PMC6521559; doi:10.1155/2019/5370847)

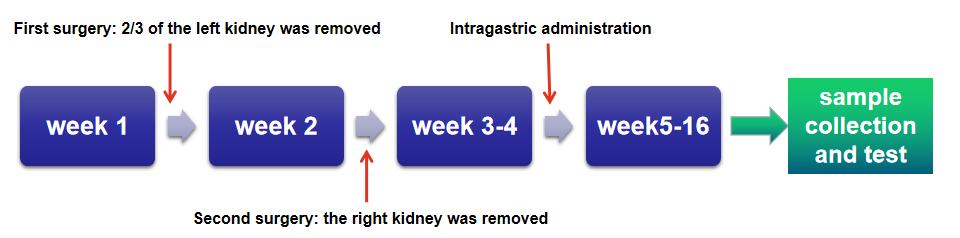

Supplement: Supplementary Materials — Figure S1: experimental design diagram. [file 5370847.f1.tif]
